# Supplementary material for: Impacts of Extreme Climate Events on Future Rice Yields in Global Major Rice-Producing Regions
Source: Int J Environ Res Public Health. 2022 Apr 7;19(8):4437. doi: 10.3390/ijerph19084437 (PMC9031651; doi:10.3390/ijerph19084437)
Supplement: Supplementary file 1 [file ijerph-19-04437-s001.zip › ijerph-1618447-supplementary.pdf]

The Supplementary Materials of "Impacts of Extreme Climate Events on Future Rice Yields in Global Major Rice-producing Regions"

This file includes:

Supplementary Table S1, Figures S1–S5

Introduction

This supporting information mainly provides the list of CMIP6 models used in this study, the spatial distribution of extreme climate events (Tx90p, Tn10p, R99p, Rx5day) in the world's four major rice-producing regions under the SSP126 and SSP585 pathways and the relationship between historical rice yield and various factors.

Table S1. Basic information and atmospheric resolution of twelve CMIP6 global climate models.

Model name	Group	Resolution
ACCESS-ESM1-5	ACCESS/Australia	1.25° × 1.875°
AWI-CM-1-1-MR	AWI/Germany	0.93° × 0.9375°
BCC-CSM2-MR	BCC/China	1.112° × 1.125°
EC-Earth3	CCCma/Canada	0.7° × 0.7°
GFDL-ESM4	GFDL/USA	1° × 1.25°
INM-CM4-8	INM/Russia	1.5° × 2°
IPSL-CM6A-LR	IPSL/France	1.27° × 2.5°
MIROC6	AORI/Japan	1.389° × 1.406°
MPI-ESM1-2-HR	MPI/Germany	0.93° × 0.9375°

MRI-ESM2-0	MRI/Japan	1.1° × 1.1°
NESM3	NESM3/China	1.85° × 1.875°
NorESM2-MM	NCC/Norway	0.94° × 1.25°

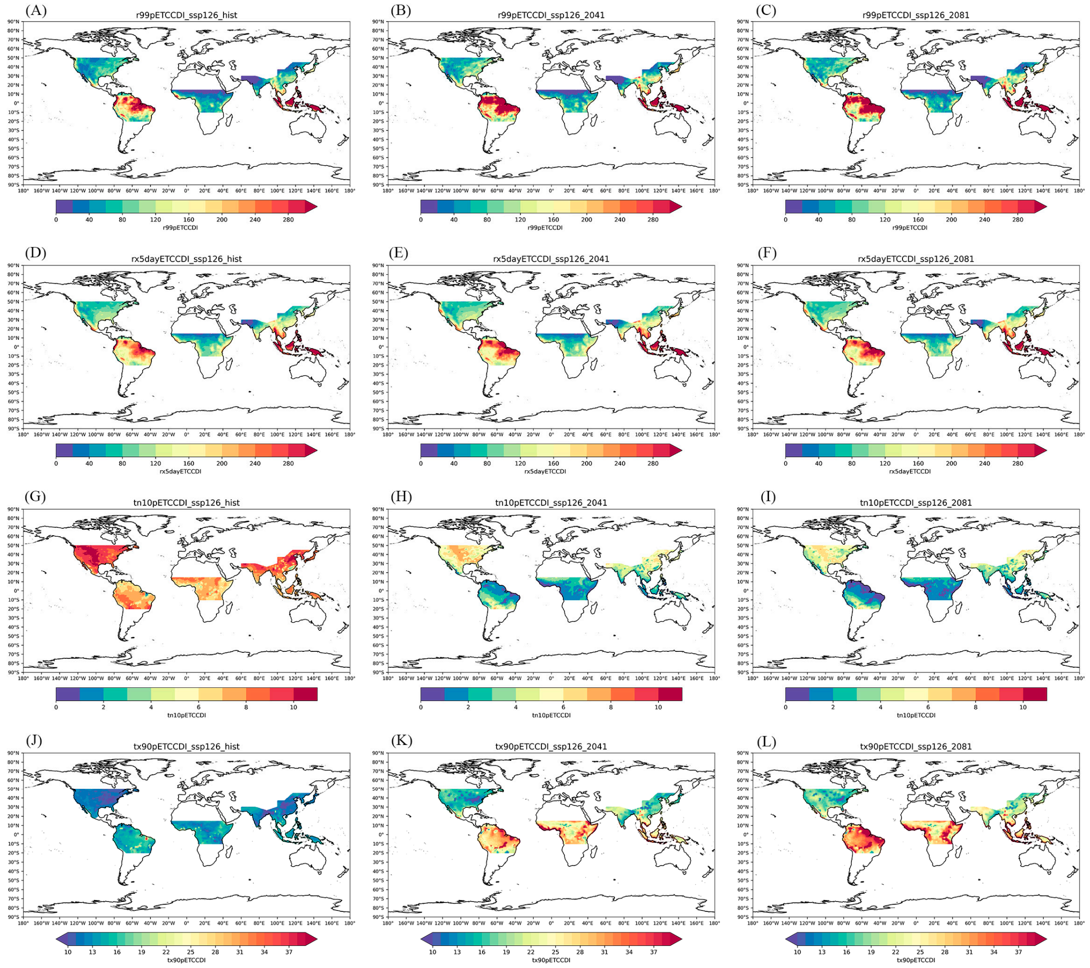

Figure S1. Spatial distribution of extreme climate events (Tx90p, Tn10p, R99p, Rx5day) in the world's four major rice-producing regions under the SSP126 pathway

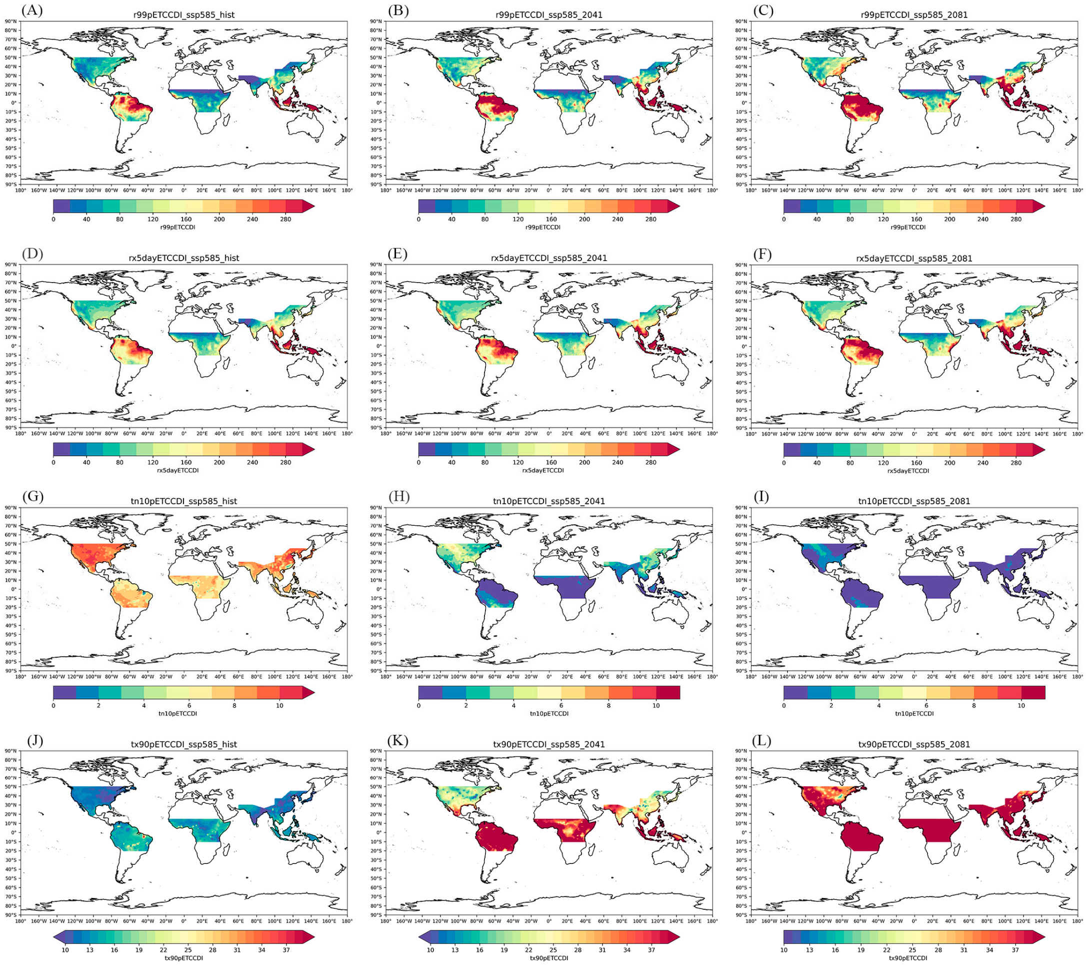

Figure S2. Spatial distribution of extreme climate events (Tx90p, Tn10p, R99p, Rx5day) in the world's four major rice-producing regions under the SSP585 pathway

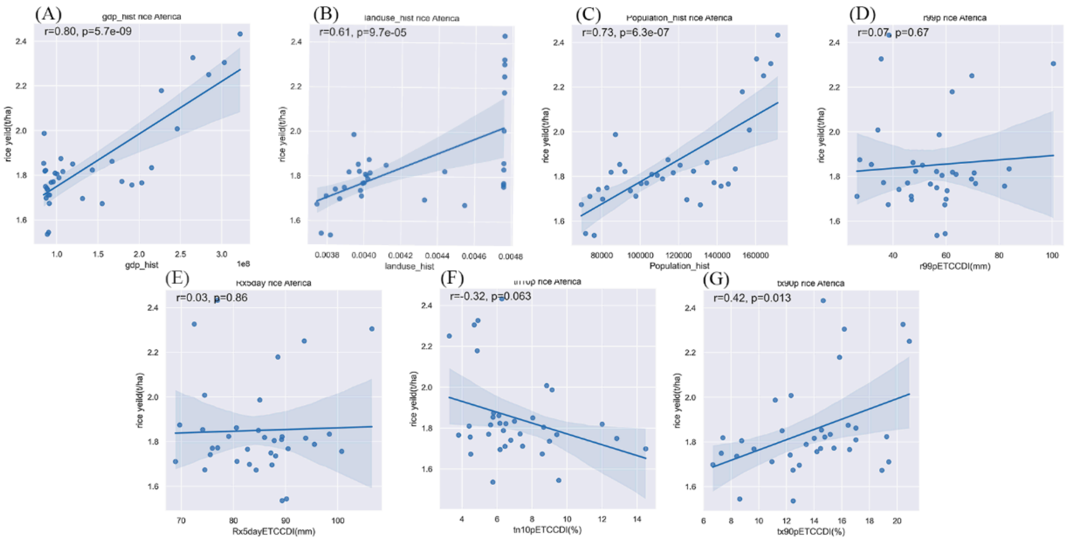

Figure S3. The relationship between extreme climate indices, economic factors and rice yield in African rice-producing region

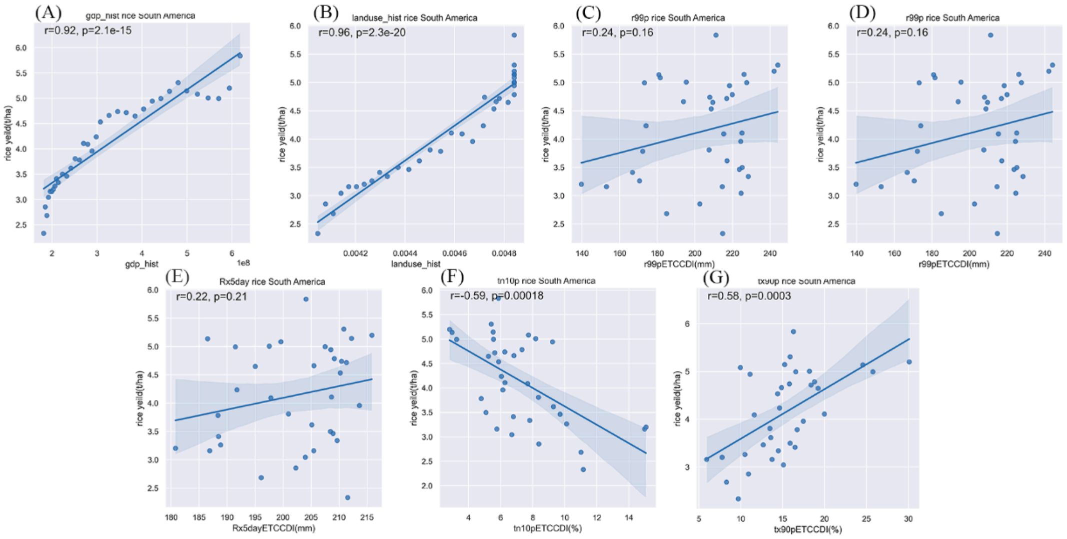

Figure S4. The relationship between extreme climate indices, economic factors and rice yield in South American rice-producing region

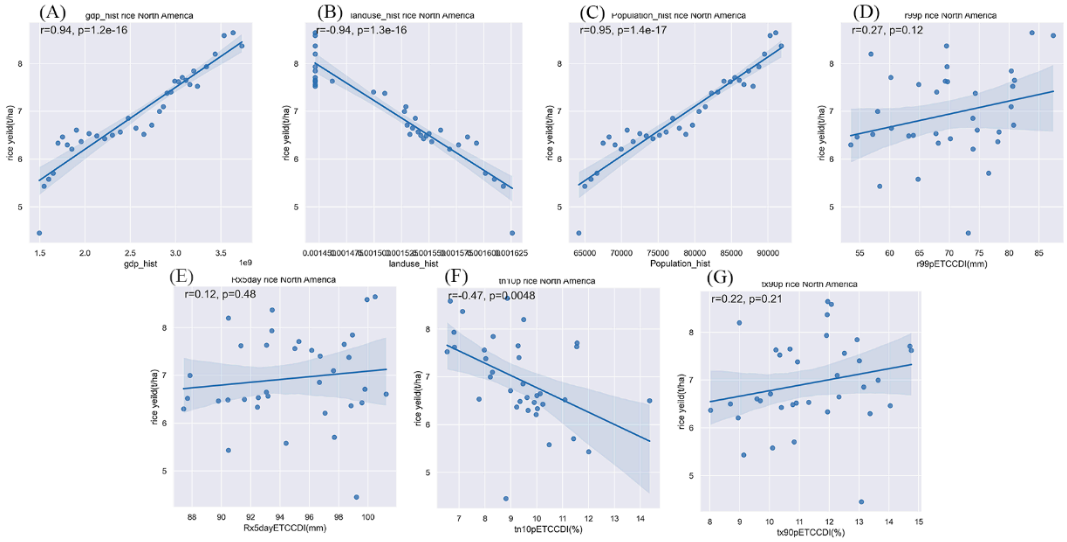

Figure S5. The relationship between extreme climate indices, economic factors and rice yield in North American rice-producing region
